# COVID-19 and Media datasets: Period- and location-specific textual data mining

**DOI:** 10.1016/j.dib.2020.106356

**Published:** 2020-09-30

**Authors:** Mathieu Roche

**Affiliations:** CIRAD, UMR TETIS, F-34398 Montpellier, France; TETIS, Univ Montpellier, AgroParisTech, CIRAD, CNRS, INRAE, Montpellier, France

**Keywords:** Corpus, Text-mining, NLP, Terminology extraction, Classification, COVID-19

## Abstract

The vocabulary used in news on a disease such as COVID-19 changes according the period [4]. This aspect is discussed on the basis of MEDISYS-sourced media datasets via two studies. The first focuses on terminology extraction and the second on period prediction according to the textual content using machine learning approaches.

## Specifications Table

**Table:** 

Subject area	Health Informatics
More specific subject area	Text-mining approaches for health and social analysis (COVID-19)
Type of data	Text
How data was acquired	Manual extraction from MEDISYS: https://medisys.newsbrief.eu/medisys/homeedition/fr/home.html
Data format	Raw: (1) Corpus for BioTex (*.txt) [repositories: *_MOOD_Corpus_BIOTEX], (2) Corpus for Weka (*.arff) [repositories: *_MOOD_Corpus_WEKA] Filtered: Terms extracted with BioTex from corpus (1) (*.csv)
Parameters for data collection	Dedicated keywords, locations and languages for selecting data from MEDISYS. Specific parameters and algorithms are applied for NLP and data mining tools (ranking measures, classification algorithms, etc.).
Description of data collection	These datasets contain a set of news articles in English, Spanish and French extracted from MEDISYS (i.e. advanced search) according dedicated criteria. A corpus (i.e. textual data) by location (UK, Spain, France) and period (March, May, July 2020) has been collected from MEDISYS. Corpora have an adapted format for BioTex (*.txt) and Weka (*.arff). Terms extracted (*.csv) with the BioTex system from these corpora are available.
Data source location	MEDISYS (open access)
Data accessibility	Repository name: Dataverse (CIRAD) Data identification number: https://doi.org/10.18167/DVN1/ZUA8MF

## Value of the Data

• This dataset is important for spatiotemporal analysis of media content regarding COVID-19. The methodology is generic and could be implemented for other study cases based on MEDISYS data.
• This data could be used by computer science scientists (NLP and data mining domains) and for social science and humanities research.
• The formats of these datasets are suitable for NLP approaches (e.g. BioTex) and data-mining tools (e.g. Weka).
• Other corpora could also be collected with the method outlined in this data paper. The code (Perl) enables the conversion of other textual data from MEDISYS in suitable formats for NLP and data-mining tools.

## 1 Data Description

These datasets contain a set of news articles in English, French and Spanish extracted from MEDISYS (i.e. advanced search) according the following criteria:

3- Keywords (at least): COVID-19, ncov2019, cov2019, coronavirus
3- Keywords (all words): mask (English), máscara (Spanish), masque (French)
3- 3 periods: March 2020, May 2020, July 2020
3- 3 locations: UK (English), Spain (Spanish), France (French)

Location-specific corpora were manually collected (copy/paste) by querying MEDISYS with the previous parameters (i.e. primary data sources). For each location, 100 snippets by period (1st, 10th, 15^th^ and 20th of each month) were built. These data were preprocessed using a dedicated Perl program followed by text-mining algorithms (see section below) in order to produce the following datasets (i.e. secondary dataset - https://doi.org/10.18167/DVN1/ZUA8MF):

3- A corpus preprocessed for the BioTex tool - https://gitlab.irstea.fr/jacques.fize/biotex_python (*.txt^1^) [∼ 900 texts];
3- The same corpus preprocessed for the Weka tool - https://www.cs.waikato.ac.nz/ml/weka/ (*.arff^2^);
3- Terms extracted with BioTex according spatiotemporal criteria (*.csv^3^) [∼ 9000 terms].

Other corpora can be collected with the same method. The code (Perl) required to preprocess textual data for terminology extraction (with BioTex) and classification (with Weka) tasks is available on Dataverse: https://doi.org/10.18167/DVN1/ZUA8MF.

The textual data acquisition phase is done manually by querying MEDISYS. The other tasks described in this paper are automatic (i.e. pre-processing, terminology extraction, classification). In future research, we plan to use RSS feeds provided by MEDISYS in order to collect data automatically.

## 2 Experimental Design, Materials and Methods

With these datasets, two experiments were conducted per location:

3- **Terminology extraction tasks using NLP approaches to highlight specific terms**. Terminology extraction is based on the BioTex system [2]. Several measures are implemented in BioTex for term ranking. The F-TFIDF-C criterion based on a combination of TF-IDF [3] and C-Value [1] were used for these datasets. TF-IDF highlights discriminative terms, while C-Value favors phrase (i.e. multiword term) extraction. The extraction results are available in the Dataverse repository associated with this study (BioTex parameters used: F-TFIDF-C measure, number of syntactic patterns: 10, 3 languages).

Table 1 presents a sample of these results, i.e. terminology selected with the word *mask* from UK_MOOD_Terms, SP_MOOD_Terms and FR_MOOD_Terms raw data. We found that the vocabulary could be period- and location-specific, but similar trends were also noted for some aspects like mandatory mask-wearing in July for all locations (e.g. *mandatory mask, mandatory mask-wearing, máscara obligatoria en comercios, máscara obligatoria, masque obligatoire*).

**Table 1:** Terms obtained with BioTex filtered with the word *mask* (*mask, máscara, masque*) and the associated rank for the 3 locations.

**UK**	**Period 1 *(March 2020)***	**Period 2 *(May 2020)***	**Period 3 *(July 2020)***
**1**	face mask (10)	coronavirus mask (19)	masks (2)
**2**	gas mask (59)	masks (40)	mask (17)
**3**	protective mask (167)	mask mess (53)	mandatory mask (237)
**4**	mask (202)	surgical mask (57)	mandatory mask-wearing (238)
**5**	masks (227)	face masks (80)	mandatory masks (239)


**Spain**	**Period 1 *(March 2020)***	**Period 2 *(May 2020)***	**Period 3 *(July 2020)***
**1**	máscara de protección (1)	máscara facial (87)	máscaras antigás con filtro (10)
**2**	máscara de snorkel (34)	máscaras quirúrgicas (152)	máscaras antigás (21)
**3**	máscara antigás (47)	máscaras (170)	máscara obligatoria en comercios (23)
**4**	máscara (80)	máscara (212)	máscara obligatoria (37)
**5**	máscara de tristeza (489)	máscara marrón (366)	diseños de máscaras (86)


**France**	**Period 1 *(March 2020)***	**Period 2 *(May 2020)***	**Period 3 *(July 2020)***
**1**	masques (3)	masques (1)	port du masque (21)
**2**	masque à abidjan (21)	masque de protection (161)	masque (32)
**3**	masques en tissu (43)	port du masque (201)	masque obligatoire (75)
**4**	masque sur le visage (86)	chant du masque (441)	masque sur le visage (77)
**5**	masques chirurgicaux (119)	fabricants de masques (442)	masque de protection (110)

3- **Classification tasks using machine learning approaches for period prediction**. Based on a vector space model representation (i.e. bag-of-words), the objective is to predict periods using machine learning approaches. Note that each month represents the class to predict with supervised learning techniques (i.e. in the ARFF files, each article has a label associated with the month).

Table 2 presents the results obtained with 3 supervised algorithms (i.e. Support Vector Machine (SMO with PolyKernel), Random Forest (bagSizePercent = 100, maxDepth = 0, numIterations = 100) and Naive Bayes) with the raw data UK_MOOD_Corpus_WEKA, SP_MOOD_Corpus_WEKA, FR_MOOD_Corpus_WEKA. Other Weka algorithms can also be used with these corpora.

**Table 2:** Classification scores (i.e. precision (P), recall (R) and F-measure (F)) using 10 cross-validations for period prediction at 3 locations (NB: Naïve Bayes, SVM: Support Vector Machine, RF: Random Forest).

**UK**	**NB**			**SVM**			**RF**		
	**P**	**R**	**F**	**P**	**R**	**F**	**P**	**R**	**F**
**Period 1**	*0.867*	*0.650*	*0.743*	*0.758*	*0.750*	*0.754*	*0.798*	*0.710*	*0.751*
**Period 2**	*0.814*	*0.350*	*0.490*	*0.598*	*0.490*	*0.538*	*0.656*	*0.420*	*0.512*
**Period 3**	*0.503*	*0.919*	*0.650*	*0.593*	*0.707*	*0.645*	*0.541*	*0.798*	*0.645*
**Accuracy**	***63.9%***	***64.9%***	***64.2%***						


**Spain**	**NB**			**SVM**			**RF**		
	**P**	**R**	**F**	**P**	**R**	**F**	**P**	**R**	**F**
**Period 1**	*0.670*	*0.770*	*0.716*	*0.735*	*0.750*	*0.743*	*0.700*	*0.700*	*0.700*
**Period 2**	*0.864*	*0.515*	*0.646*	*0.685*	*0.636*	*0.660*	*0.768*	*0.535*	*0.631*
**Period 3**	*0.637*	*0.798*	*0.709*	*0.702*	*0.737*	*0.719*	*0.620*	*0.808*	*0.702*
**Accuracy**	***69.4%***	***70.8%***	***68.1%***						


**France**	**NB**			**SVM**			**RF**		
	**P**	**R**	**F**	**P**	**R**	**F**	**P**	**R**	**F**
**Period 1**	*0.772*	*0.780*	*0.776*	*0.812*	*0.820*	*0.816*	*0.798*	*0.830*	*0.814*
**Period 2**	*0.759*	*0.850*	*0.802*	*0.775*	*0.790*	*0.782*	*0.761*	*0.860*	*0.808*
**Period 3**	*0.862*	*0.750*	*0.802*	*0.845*	*0.820*	*0.832*	*0.928*	*0.770*	*0.842*
**Accuracy**	***79.3%***	***81.0%***	***82.0%***

## Ethics Statement

The author confirms compliance with the ethical policies of the journal, as noted on the journal's author guidelines page. No ethical approval was required because this study did not involve any experimental protocol on humans or animals, and only open source online data were used. This work is based on a sample of data from MEDISYS (public site) that is an open access system available for all users^4^.

## Declaration of Competing Interest

The author declares no conflicts of interest.

## Appendix Supplementary materials

Image, application 1

## Footnotes

4 https://external.ecdc.europa.eu/EI_Tutorial/modulo01/unita02/html/MedISys_tutorial.pdf

## Footnotes

1 Each article (line) is separated by a blank line.

2 https://www.cs.waikato.ac.nz/∼ml/weka/arff.html

3 Each line includes the rank, term and presence (i.e. value = 1) of the term in UMLS (Unified Medical Language System) and the BioTex score based on the F-TFIDF-C measure.
